# Undetectable or subtherapeutic serum levels of antipsychotic drugs preceding switch to clozapine

**DOI:** 10.1038/s41537-020-0107-7

**Published:** 2020-07-17

**Authors:** Lennart Kyllesø, Robert Løvsletten Smith, Øystein Karlstad, Ole A. Andreassen, Espen Molden

**Affiliations:** 1 Center for Psychopharmacology, Diakonhjemmet Hospital, Oslo, Norway; 2 Department of Chronic Diseases and Ageing, Norwegian Institute of Public Health, Oslo, Norway; 3 NORMENT Centre, Division of Mental Health and Addiction, University of Oslo and Oslo University Hospital, Oslo, Norway; 4 Section for Pharmacology and Pharmaceutical Biosciences, Department of Pharmacy, University of Oslo, Oslo, Norway

**Keywords:** Schizophrenia, Schizophrenia, Psychosis

## Abstract

Adequate antipsychotic treatment intensity is required for defining treatment-resistant schizophrenia (TRS) and justifying clozapine treatment. We investigated the occurrence of undetectable or subtherapeutic serum levels of oral antipsychotics preceding switch to clozapine as an endpoint of TRS. For patients starting clozapine, 12-month retrospective reviews of antipsychotic serum concentration measurements were performed in a Norwegian therapeutic drug monitoring (TDM) database from 2005 to 2017. Undetectable levels in high-sensitive analytical assays defined ‘no drug use’, while levels <50% of the lower reference range defined ‘subtherapeutic use’. Similar data were collected for patients switching to long-acting injectable (LAI) antipsychotics, as a reference of ‘no or subtherapeutic drug use’. Nineteen of 353 patients initiating clozapine (5.4%) had a recent history of undetectable antipsychotic drug levels compared to 91 of 1048 (8.7%) initiating LAI. Another 19 patients starting clozapine (5.4%) had recent events of subtherapeutic levels. In conclusion, the present retrospective study may indicate that every 10th patient starting clozapine has a recent history of undetectable or subtherapeutic serum levels of oral antipsychotics. The clinical implications of the present study for the assessment of TRS should be investigated prospectively in further studies.

## Introduction

Schizophrenia is regarded as the most severe mental illness, both in terms of patient suffering and societal costs^1–3^. Affecting millions of people worldwide, schizophrenia is characterized by symptoms such as hallucinations, delusions, lack of motivation and social withdrawal^1–3^. Chronic use of antipsychotic medication is regarded as necessary in the treatment of schizophrenia^4^, but about one-third of the patients respond insufficiently to antipsychotics^5,6^. These patients are classified as ‘treatment resistant’ or ‘treatment refractory’. The term ‘treatment-resistant schizophrenia’ (TRS) is defined as a ‘lack of satisfactory clinical improvement despite the use of adequate doses of at least two different antipsychotic agents, including an atypical antipsychotic agent, prescribed for adequate duration’^7–9^. However, for a better understanding of the underlying causes of TRS and for optimizing treatment in these patients, it is crucial to discriminate pseudo-resistance (inadequate treatment intensity) from a true TRS (ineffective treatment)^10^.

The atypical antipsychotic clozapine is currently the only drug licenced for TRS, and 60–70% of the patients obtain a clinically relevant response after switching to clozapine^11,12^. The characterization of clozapine as the most effective drug in treating schizophrenia is supported by meta-analyses by Leucht et al.^13^, Masuda et al.^14^ and Huhn et al.^15^, showing its clinical superiority compared to all other oral antipsychotics. It has also been shown that clozapine treatment provides a significantly lower risk of all-cause mortality versus other oral antipsychotics^16^. Tiihonen et al.^17^ recently reported that clozapine and long-acting injectable (LAI) antipsychotics had the highest reduction in the risk of rehospitalization or any treatment failure in schizophrenia. However, because of the risk of serious adverse events like agranulocytosis and tonic-clonic seizures, clozapine is only labelled for use in patients with TRS^18^, although national prescribing guidelines differ somewhat on this point^7,12^. Furthermore, it is required that the exposure to antipsychotic drugs has been adequate to confirm a true TRS, and hence qualify for clozapine use^9^.

Interindividual pharmacokinetic differences and adherence issues may lead to variations in drug serum levels^19,20^. A group of German psychiatrists has developed and published the Consensus Guidelines for Therapeutic Drug Monitoring (TDM) in Neuropsychopharmacology (AGNP) to standardize target concentration ranges of psychotropic drugs^19^. Subtherapeutic exposure to antipsychotic drugs, indicated by serum levels well below the target range, may occur for various reasons. Treatment nonadherence is often regarded as a frequent cause^21,22^. Poor adherence is a known problem in chronic conditions requiring long-term treatment^23,24^, and the nonadherence rates of antipsychotic drugs are reported to range from 40% to 70% among patients with schizophrenia^9,25–27^. Even though treatment nonadherence may appear as TRS, Howes et al.^9^ found that only 5% of studies on TRS had, in fact, assessed adherence. This demonstrates the need of a deeper understanding of the underlying causes of initiating clozapine treatment, which have clinical safety implications.

Recently, McCutcheon et al.^28^ reported in a population of 99 patients that about one-third with clinically defined treatment-resistance, but not prescribed clozapine, had subtherapeutic plasma levels of antipsychotic drugs. This indicates that the TRS diagnosis in clinical practice is often not compliant with the consensus recommendation^9^. However, it is necessary with more research in larger patient populations to obtain firm data on the extent of subtherapeutic plasma levels of ongoing antipsychotic treatment when TRS is diagnosed, and preferably in patients where the psychiatrists have decided to prescribe clozapine as a definite endpoint of clinically interpreted TRS.

In order to gain knowledge on the occurrence of insufficient treatment intensity preceding clozapine use, we investigated the frequency of undetectable or subtherapeutic serum levels of oral antipsychotic drugs before starting clozapine in a large patient population with longitudinal TDM profiles. In addition, we compared the frequency of undetectable or subtherapeutic serum concentrations of antipsychotic drugs in patients prior to prescribing of clozapine versus LAI antipsychotics, which is the recommended treatment alternative in cases of nonadherence.

## Results

### Characteristics of the included patients

In total, 353 patients with 1364 concentrations measurements of oral antipsychotics in their TDM records before starting clozapine were included in the study. Out of these, 251 (71.1%) were inpatients at the time of most recent blood sampling prior to initiation of clozapine. In comparison, those who started LAI antipsychotics comprised of 1048 patients (2758 concentration measurements), of whom 746 (71.2%) were inpatients.

In the clozapine group, there were no significant differences in demographic characteristics (age, sex) between patients with and without a recent history of undetectable or subtherapeutic serum levels prior to starting clozapine (*p* > 0.5, Table 1), but the observed proportion of inpatients was higher in the subgroup with undetectable or subtherapeutic compared to patients with serum concentrations in the target range, i.e. 84.2% versus 69.5%, respectively (*p* = 0.06, Table 1). According to the information on the requisition forms, the daily prescribed doses of the antipsychotics preceding clozapine were on average 2.5-fold higher than the minimum therapeutic dose recommendations in schizophrenia treatment (95% CI: 2.3-fold to 2.7-fold, Table 1). Further, the prescribed daily doses relative to the drugs’ minimum schizophrenia dose recommendations were not different between patients with undetectable or subtherapeutic vs. those with therapeutic serum concentrations of antipsychotics drugs before initiating clozapine (*p* = 0.41, Table 1). The prescribed antipsychotic doses at the undetectable or subtherapeutic events ranged from 100% to 500% of the lower recommended doses (median 150%, data not shown).

**Table 1:** Characteristics of the included patients according to serum level history prior to initiating therapeutic drug monitoring of clozapine.

Characteristics	Total, *n* = 353	Undetectable/subtherapeutic, *n* = 38	Therapeutic, *n* = 315	*p*
Women, *n* (%)	129 (36.5)	12 (31.6)	117 (37.1)	0.6
Age, years, mean (SD)	33.8 (11.6)	34.7 (9.5)	33.6 (11.8)	0.6
Inpatients, *n* (%)	251 (71.1)	32 (84.2)	219 (69.5)	0.06
Analysed samples of antipsychotics in TDM records, mean (SD)	3.9 (3.5)	6.6 (4.7)	3.6 (3.2)	<0.001
TDM history of multiple antipsychotics, *n* (%)	205 (58.1)	34 (89.5)	171 (54.3)	<0.001
Relative dose at final TDM prior to clozapine, fold/mean (SD)	2.51 (1.97)	2.26 (1.39)	2.54 (2.03)	0.41

Age, patient age at date of first registered TDM of clozapine. Subtherapeutic patients were defined as a person with at least one subtherapeutic or undetectable sample of antipsychotic drug (and metabolite, if applicable) in a TDM sample during the observation period, despite indicated dose in the submitted requisition form. Undetectable was defined as absence/below Lower Limit Of Detection (LLOD) of the prescribed drug (both drug and metabolite if applicable) ordered for TDM by the physician, in the respective patient’s blood sample submitted for concentration analysis. Age and number of samples per patient (during observation period) were compared between patients with and without subtherapeutic serum level events using Student’s *T*-test. Reference ranges and prescribed doses relative to lower recommended therapeutic dose in schizophrenia were adapted from the AGNP 2017 consensus guidelines^19^.

*SD* standard deviation, *TDM* therapeutic drug monitoring.

Among the patients who had undetectable or subtherapeutic levels before starting clozapine, 89.5% had measured two or more unique (different) oral antipsychotics during the retrospective observation period, regardless of timing, as compared to 54.3% among patients with therapeutic serum levels (*p* < 0.001, Table 1).

### Rates of undetectable and subtherapeutic serum levels prior to initiation of clozapine

Among the included patients, 10.8% (*n* = 38, 95% CI: 7.7–14.5; Table 2) had a history of at least one TDM analysis with undetectable or subtherapeutic serum levels of oral non-clozapine antipsychotics within the 12-month period prior to initiation of clozapine TDM, whereof 5.4% (*n* = 19, 95% CI: 0.65–17.7) of the patients had undetectable concentrations (Table 2). In comparison, for those initiating TDM of LAI, the patient proportion with a recent history of undetectable or subtherapeutic levels was 17.9% (*n* = 188, 95% CI: 15.7–20.4), whereof concentrations were undetectable in 8.7% (*n* = 91, 95% CI: 4.9–13.5) of the patients (Table 2). The median period from the undetectable or subtherapeutic TDM event to first recorded TDM event of clozapine was 138 days (Interquartile range 70–255 days). For patients who switched to LAI, the median period was 113 days (Interquartile range 48–187 days).

**Table 2:** Rates of undetectable and subtherapeutic serum levels among patients within 12 months prior to initiating therapeutic drug monitoring of clozapine or LAI antipsychotics.

Patient group	Undetectable/subtherapeutic, combined	Undetectable
Clozapine patients, *n* (%, 95% CI)	38 (10.8, 7.7–14.5)	19 (5.4, 0.65–17.7)
LAI patients, *n* (%, 95% CI)	188 (17.9, 15.7–20.4)	91 (8.7, 4.9–13.5)

Subtherapeutic patients were defined as a person with at least one subtherapeutic sample of antipsychotic drug (and metabolite, if applicable) in a TDM sample during the observation period, despite indicated dose in the submitted requisition form. Subtherapeutic levels were defined as a serum concentration below 50% of the lower reference range boundary. Undetectable was defined as absence/below Lower Limit Of Detection (LLOD) of the prescribed drug (both drug and metabolite if applicable) ordered for TDM by the physician, in the respective patient’s blood sample submitted for concentration analysis. Reference ranges and prescribed doses relative to lower recommended therapeutic dose in schizophrenia were adapted from the AGNP 2017 consensus guidelines^19^.

*LAI* long-acting injectables, *TDM* therapeutic drug monitoring.

Among the patients changing antipsychotic treatment, 33% (*n* = 13, 95% CI: 19.1–50.2) and 41% (*n* = 92, 95% CI: 34.6–47.8, *p* = 0.383) of the cases with undetectable or subtherapeutic concentrations of non-clozapine antipsychotics occurred at the last TDM event before switching to clozapine or LAI, respectively. Table 3 provides an overview of the frequencies of the various non-clozapine antipsychotic drugs measured in patients with versus without a history of undetectable or subtherapeutic serum levels prior to starting clozapine TDM (Table 3). For all the antipsychotics, there were no significant differences in prescribed doses in patients with versus without a history of undetectable or subtherapeutic serum levels before initiation of clozapine (*p* > 0.2; data not shown). Drug metabolic rates (metabolite to parent ratio, MPR) of the agents with metabolite analyses included in the TDM assays, i.e. risperidone and aripiprazole, did not differ between patients with or without recent TDM events of undetectable or subtherapeutic antipsychotic concentrations (*p* > 0.5, data not shown).

**Table 3:** Frequency of various oral antipsychotics analysed in blood samples during the whole observation period prior to initiating therapeutic drug monitoring of clozapine in patients with undetectable or subtherapeutic versus therapeutic TDM events.

Antipsychotic drug	Undetectable/subtherapeutic, *n* (%)	Therapeutic, *n* (%)	*p*
Total samples	226 (100)	1138 (100)	–
Amisulpride	1 (0.4)	43 (3.9)	n.a.
Aripiprazole	19 (8.4)	102 (9.0)	0.90
Chlorprothixene	2 (0.9)	22 (1.9)	n.a.
Flupentixol	3 (1.3)	10 (0.9)	n.a.
Haloperidol	6 (2.7)	20 (1.8)	n.a.
Levomepromazine	3 (1.3)	14 (1.2)	n.a.
Olanzapine	62 (27.4)	367 (32.3)	0.16
Perphenazine	13 (5.8)	34 (3.0)	0.06
Quetiapine	52 (23.0)	263 (23.1)	1.0
Risperidone	22 (9.7)	112 (9.8)	1.0
Sertindole	10 (4.4)	14 (1.2)	0.003
Ziprasidone	20 (8.9)	49 (4.3)	0.007
Zuclopenthixol	13 (5.8)	88 (7.7)	0.33

Subtherapeutic samples were defined as a TDM sample indicating subtherapeutic levels of antipsychotic drug (and metabolite, if applicable) during the observation period, despite indicated dose in the submitted requisition form. Subtherapeutic levels were defined as a serum concentration below 50% of the lower reference range boundary, above minimum therapeutic doses. Undetectable was defined as absence/below Lower Limit Of Detection (LLOD) of the prescribed drug (both drug and metabolite if applicable) ordered for TDM by the physician, in the respective patient’s blood sample submitted for concentration analysis. Reference ranges and minimum therapeutic doses were adapted from the AGNP 2017 consensus guidelines^19^.

*TDM* therapeutic drug monitoring.

## Discussion

The present descriptive study may indicate that every 10th patient starting clozapine has a recent history of undetectable or subtherapeutic serum levels of oral non-clozapine antipsychotics. Thus, as clozapine is usually prescribed for TRS, the potential occurrence of insufficient serum concentrations of oral non-clozapine antipsychotics should be considered as a relevant aspect to include in the clinical assessment of treatment failure in patients with schizophrenia. Further, the findings support that TDM may be valuable as an assisting tool in the clinical evaluation of the underlying reason(s) for treatment failure of antipsychotic drugs.

TRS is the main indication for clozapine use, and hence clozapine initiation is an indirect measure of this diagnosis. This study investigated the occurrence of undetectable or subtherapeutic serum levels in patients using initiation of clozapine TDM as an objective measure of TRS. However, a related study by McCutcheon et al.^28^ on 99 patients diagnosed with TRS by other criteria, reported that 35% had subtherapeutic serum levels in TDM samples of one or more antipsychotics. Among these, one-third of the subtherapeutic levels represented undetectable concentrations in the TDM samples, which is in line with the results from our present study, where a substantially larger patient population (*n* = 353) with TRS was included. Overall, the two studies therefore suggest that insufficient treatment intensity is quite common as a potential issue in the interpretation of TRS.

Pharmacokinetic factors and adherence issues are typical sources of subtherapeutic serum levels, while undetectable levels are unequivocally defined as nonadherence. To better understand the degree of undetectable antipsychotic serum levels (i.e. complete nonadherence) in patients with prescribed doses compliant with schizophrenia, we included a group of patients starting LAI antipsychotics. Nonadherence to oral antipsychotic drugs is a major indication for LAI use. Interestingly, 8.7% of the patients starting LAI antipsychotics had a history of undetectable serum levels in the retrospective 12-month observation period, which was only 1.6-fold as frequent as for the patients starting clozapine. This may indicate that the psychiatrists’ decision of prescribing clozapine to possibly nonadherent patients does not fully comply with the Treatment Response and Resistance In Psychosis (TRRIP) consensus criteria to thoroughly assess adherence prior to initiation of clozapine^9^. Recently, Kane and Correll^29^ stated that prescribing of LAI antipsychotics should be preferred in nonadherent patients, and use of LAI is associated with less severe side effects and with clinical effectiveness approaching that of clozapine in preventing all-cause hospitalization^17,30^. The TRRIP criteria also recommend prescribing a LAI before defining TRS to exclude potential nonadherence as reason of treatment failure^9^.

According to the TRRIP criteria, a duration of at least 6 weeks of adequate antipsychotic dosing/exposure in monotherapy is required before defining a failed drug trial^9^. Therefore, an absolute minimum of 12 weeks applies to two failed trials. In the present study, the median time period between the TDM event of undetectable or subtherapeutic concentrations of non-clozapine antipsychotics and the first TDM event of clozapine were about 20 weeks. It is therefore a theoretical possibility that two separate drug treatments failed during this time period. However, the limited difference in the frequency of undetectable serum concentrations at the final TDM event prior switching to clozapine vs. LAI (33% vs. 41%) indicates that clozapine is quite often initiated in patients with recent nonadherence. In addition, when including the duration required for up-titration and down-titration of doses during an antipsychotic drug switch, it seems unlikely that failed treatments of two separate non-clozapine antipsychotics occurred between the most recent event of undetectable or subtherapeutic concentrations and the first TDM event of clozapine.

Poor adherence is indeed a major clinical issue and a constraint to optimal outcomes in the treatment of patients with schizophrenia^26,30–33^. There is also growing concern regarding the escalating use of health care resources among poorly managed patients with schizophrenia, whose persistent symptoms can be linked to nonadherence issues^27,34^. There are several methods for measuring nonadherence^35–37^. In the present study, we applied a conservative approach by classifying nonadherence of antipsychotic drugs as undetectable drug levels in blood samples. This provides a robust and clinically relevant measure, as undetectable or very low drug levels in blood samples obviously may result in treatment failure and acute symptom exacerbation. In contrast, most other methods define nonadherence inconsistently and broadly, with a mix of situations ranging from minor discrepancies between prescribed and actual time of dose intake, occasionally missed doses, to major events of persistent antipsychotic discontinuation^19,32,35,36^. This is the reason why our 5% estimate of *undetectable* serum levels as a measure of complete nonadherence in the present study is substantially lower than those of previous studies, where the widely defined nonadherence rates typically range from 40% to 70%^25–27^.

With the low detection limits of the analytical assays as the underlying premise of defining undetectable serum levels as nonadherence in the present study, undetectable levels strongly indicate that a patient had been nonadherent for a period of several days to weeks before the TDM analysis. Many studies have associated poor adherence with an increased risk of relapse and hospitalization^4,17,38,39^. Thus, it is of little doubt that the risk of symptom relapse and potential hospitalization is high among the patients with undetectable serum levels in the present study. It is also likely that patients with serum concentrations <50% of the lower therapeutic range will have increased risk of symptom relapse. The higher proportion of inpatients than outpatients with subtherapeutic or undetectable concentrations in the present study support the clinical relevance of poor adherence as a risk factor for hospitalization.

Subtherapeutic concentrations of antipsychotics may also occur as a result of high clearance/ultra-rapid metabolism^21^. This implies a theoretical risk of misinterpreting low concentration as nonadherence. However, in the study population, the drug metabolism rates of aripiprazole and risperidone, two of the most commonly used antipsychotic drugs in schizophrenia, did not differ between patients with subtherapeutic vs. therapeutic concentration, providing strong evidence that ultra-rapid drug metabolism did not confound the nonadherence classification of patients in the current study population. Furthermore, according to standard TDM routine, blood samples for serum concentration measurements are generally collected between 12 and 24 h after the last intended drug intake. Thus, it is unlikely that pharmacokinetic factors or issues related to sampling time affected the interpretations of undetectable subtherapeutic serum concentrations. Instead, complete or partial nonadherence are probably representing the origin of concentrations being (i) undetectable in the analytical assays and (ii), below 50% the lower therapeutic boundary of the target reference ranges, respectively.

Despite that undetectable and subtherapeutic antipsychotic serum levels increase the risk of therapeutic failure, an important limitation of the present study is the lack of clinical data, including the assessments made by the physicians underlying the decisions on change in drug prescribing and the treatment outcomes after switching to clozapine or LAI. Further, the specific indications for requesting TDM analyses were not known. Therefore, it could not be excluded that the study population was overrepresented by nonadherence patients, but this potential bias would be similar for patients switching to clozapine or LAI. Finally, an important notion is that we interpreted prescribing and TDM of clozapine as TRS. Although this serves as a conservative measure of TRS, clozapine may also be prescribed in patients with psychotic symptoms of Parkinson’s disease or tardive dyskinesias induced by non-clozapine antipsychotics. However, the prescribing of on average more than two-fold the lowest recommended schizophrenia doses of non-clozapine antipsychotics before switching to clozapine, suggest that the majority of patients had a diagnosis of schizophrenia.

In summary, the results from this observational, retrospective study may indicate that every 10th patient with schizophrenia starting clozapine have a recent history of undetectable or subtherapeutic serum levels of one or more non-clozapine antipsychotic drugs. The potential under-treatment with non-clozapine antipsychotics in these patients may not fully comply with the guideline criteria for clozapine initiation. The clinical implications of the present findings for the assessment of TRS, and the possible role of TDM as a tool to optimize the treatment effects of non-clozapine antipsychotic drugs, should be investigated prospectively in future studies.

## Methods

### Study population and setting

Patients (16–64 years) with schizophrenia subjected to TDM of oral antipsychotics prior to starting clozapine, were included from the TDM database at the Center for Psychopharmacology, Diakonhjemmet Hospital, Oslo, Norway, during the period January 2005 throughout December 2017. In addition, patients treated with oral non-clozapine antipsychotics prior to initiating LAI antipsychotics were included as a group with an expected high frequency of undetectable or subtherapeutic concentrations.

TDM is routinely used as a basis for tailoring drug treatment and clinical follow-up in Norwegian outpatient and inpatient settings, and the analyses are reimbursed by the national health service. The main rationale for ordering TDM analyses of psychotropic drugs is to assess lack of treatment effect, and dose-dependent side effects^19^. Center for Psychopharmacology annually performs TDM analyses of psychotropic drugs in serum samples from about 40,000 patients. Approximately 25% of the blood samples submitted for measuring antipsychotic drugs are from inpatients.

In the present study, patients were included regardless of clinical setting. The inclusion criteria were (i) a recorded TDM history of at least one oral antipsychotic medication within 12 months prior to initiating clozapine (or LAI antipsychotics), (ii) information on prescribed daily doses of recent drugs, and (iii) daily doses of respective oral antipsychotics required to be compliant with the diagnosis of schizophrenia (Supplementary Table 1). The oral antipsychotics included from the retrospective TDM records were one or more of the following agents: *amisulpride, aripiprazole, chlorprothixene, flupenthixol, haloperidol, levomepromazine, olanzapine, perphenazine, quetiapine, risperidone, sertindole, ziprasidone and zuclopenthixol*. This above information, as well as sex, age and inpatient/outpatient status, was retrieved from the patients’ TDM files.

The study was approved by the Regional Committee for Medical and Health Research Ethics (approval no. 2014/1185) and the Hospital Investigational Review Board. As the study was solely based on historical data, patient consent was not required.

### Definitions of undetectable and subtherapeutic serum concentrations

In the study, the therapeutic reference concentration ranges of the various antipsychotic drugs were drawn from the AGNP 2017 consensus guidelines^19^. The occurrence of undetectable or subtherapeutic serum concentrations prior to initiating clozapine treatment was identified from the respective patients’ TDM profiles of antipsychotic drugs within 12 months prior to the first TDM event of clozapine. Patients with undetectable serum concentrations of antipsychotic drugs during prescribing of doses compliant with schizophrenia diagnosis were identified in the study population, where ‘undetectable’ according to the analytical methods sensitivities implied concentrations below the Lower Limit of Detection (LLOD) (see Supplementary Table 1 for details on LLOD related to the lower therapeutic serum concentration range for each of the drugs included). Patients with serum concentrations <50% of the lower boundary of the AGNP reference ranges of the various antipsychotic drugs were correspondingly identified and defined as ‘subtherapeutic’. Information about the prescribed antipsychotic doses were collected from the TDM requisition forms filled out by the physicians ordering the analyses. Official prescribing information for each drug was used to verify dosing compliant with schizophrenia diagnosis.

### Serum concentration analyses of antipsychotic drugs and metabolites

The liquid chromatography tandem mass spectrometry (LC–MS/MS) assays used for serum concentration determination of the antipsychotics were validated and certified for routine TDM according to the bioanalytical requirements of the US Food and Drug Administration (FDA)^40^. Serum concentration measurements of metabolites were also included in the analytical assays for aripiprazole (dehydroaripiprazole) and risperidone (9-hydroxyrisperidone). During the time course of the retrospective data collection, the analytical assays had been slightly modified because of renewal of the analytical instrumentation, but all modifications were cross-validated according to standard criteria defined by the FDA^40^.

Briefly, the current ultra-performance LC–MS/MS (UPLC MS/MS) method, measuring all the antipsychotic drugs and metabolites in the same assay, was performed by chromatographic separation on an Acquity UPLC BEG shield RP18 column (1.7 µm, 1.0 × 100 mm; Waters, Milford, MA, USA) with gradient elution at 40 °C using a mobile phase mix of ammonium acetate buffer (pH = 4.8) and acetonitrile (18–45%). The assays’ LLODs for the various antipsychotic drugs were between 80% and 97% less than the lower boundary of their respective therapeutic concentration ranges (Supplementary Table 1). When the MS/MS signal of an antipsychotic drug was below its LLOD, this strongly suggested that the respective agent had not been used for several days or weeks prior to blood sampling.

### Measures and statistics

The main outcome measure was the frequency of patients with at least one undetectable or subtherapeutic serum concentration analysis within 12 months prior to the first TDM event of clozapine (or LAI). For the simple comparison of sex, age, clinical setting and frequency of antipsychotic TDM among the study groups, Student’s *T*-test and Fisher’s exact test were used for continuous and dichotomous variables, respectively. For patients treated with antipsychotics comprising metabolite measurements in the TDM assays, i.e. risperidone and aripiprazole, metabolic rates (metabolite to parent compound ratio—MPR) were compared between patients with and without subtherapeutic histories by the use of a mixed model analysis with restricted maximum likelihood (REML), allowing for inclusion of multiple samples per patient.

All statistical analyses were two-sided, and a *p*-value of < 0.05 was defined as statistically significant. Stata v.15.1 (StataCorp, TX, USA) software was used for the statistical analyses.

### Reporting summary

Further information on experimental design is available in the Nature Research Reporting Summary linked to this paper.

## Supplementary information

Supplementary table for manuscript

Reporting summary

## Data Availability

The data that supports the findings of this study are available upon reasonable request from the corresponding author. The data are not publicly available due to privacy and ethical restrictions.
