# Supplementary material for: Undetectable or subtherapeutic serum levels of antipsychotic drugs preceding switch to clozapine
Source: NPJ Schizophr. 2020 Jul 17;6:17. doi: 10.1038/s41537-020-0107-7 (PMC7367852; doi:10.1038/s41537-020-0107-7)
Supplement: Supplementary file 1 — Supplementary table for manuscript [file 41537_2020_107_MOESM1_ESM.pdf]

Supplementary information

Supplementary table 1. Detailed information about minimum recommended doses, licenced maximum doses, therapeutic serum concentration ranges and the analytical assay's lower limit of detections (LLODs) for the antipsychotic drugs.

**Agent**	**Minimum recommended daily dose in schizophrenia (mg/day) †**	**Licenced maximum daily dose (mg/day) †**	**Therapeutic serum concentration range (nmol/L) ‡**	**Proportion LLOD of the lower therapeutic serum concentration range**
Aripiprazole	15	1200	200-1300	0.05
Amisulpride	400	30	100-1500	0.20
Flupentixol	2	450+	1-15	0.10
Haloperidol	2	18	2-25	0.10
Chlorprotixene	100	20	10-100	0.10
Levomepromazine	100	1000	10-300	0.10
Olanzapine	10	20	30-200	0.07
Perphenazine	12	24	1-7	0.1
Quetiapine	300	750	50-700	0.16
Risperidone	2	16	20-120	0.02
Sertindol	12	24	30-200	0.13
Ziprasidone	40	160	40-300	0.05
Zuclopentixol	10	150	5-35	0.08

†For schizophrenia, as suggested by AGNP 2017 consensus guidelines ‡ According to AGNP 2017 guidelines
